# Reduced Intake of Dietary Tryptophan Improves Beneficial Action of Budesonide in Patients with Lymphocytic Colitis and Mood Disorders

**DOI:** 10.3390/nu15071674

**Published:** 2023-03-30

**Authors:** Cezary Chojnacki, Anita Gąsiorowska, Tomasz Popławski, Aleksandra Błońska, Paulina Konrad, Radosław Zajdler, Jan Chojnacki, Janusz Blasiak

**Affiliations:** 1 Department of Clinical Nutrition and Gastroenterological Diagnostics, Medical University of Lodz, 90-647 Lodz, Poland; cezary.chojnacki@umed.lodz.pl (C.C.); aleksandra.blonska@umed.lodz.pl (A.B.); paulina.konrad@umed.lodz.pl (P.K.); 2 Department of Gastroenterology, Medical University of Lodz, 92-213 Lodz, Poland; anita.gasiorowska@umed.lodz.pl; 3 Department of Pharmaceutical Microbiology and Biochemistry, Medical University of Lodz, 92-215 Lodz, Poland; tomasz.poplawski@umed.lodz.pl; 4 Department of Computer Science in Economics, University of Lodz, 90-237 Lodz, Poland; radoslaw.zajdler@umed.lodz.pl; 5 Department of Molecular Genetics, University of Lodz, 90-236 Lodz, Poland

**Keywords:** lymphocytic colitis, tryptophan, tryptophan metabolism, anxiety and depression, dietary intervention, food intake control

## Abstract

Lymphocytic colitis (LC) is a gastrointestinal (GI) tract disease with poorly known pathogenesis, but some environmental and lifestyle factors, including certain dietary components, may play a role. Tryptophan is an essential amino acid, which plays important structural and functional roles as a component of many proteins. It is important in the development and maintenance of the body, in which it is metabolized in two main pathways: kynurenine (KYN) and serotonin. In this work, we explored the effect of reducing of TRP in the diet of patients with LC with mood disorders. We enrolled 40 LC patients who had a normal diet, 40 LC patients with the 8-week diet with TRP content reduced by 25% and 40 controls. All LC patients received budesonide at 9 mg per day, and the severity of their GI symptoms was evaluated by the Gastrointestinal Symptoms Rating Scale. Mood disorders were evaluated by the Hamilton Anxiety Rating Scale (HAM-A) and the Hamilton Depression Rating Scale (HAM-D). The concentration of TRP and its metabolites, 5-hydroxyindoleacetic acid (5-HIAA), kynurenine (KYN), kynurenic acid (KYNA) and quinolinic acid (QA), in urine were determined. Budesonide improved the GI and mental states of LC patients, and the diet with reduced TRP content further amended these symptoms. Dietary intervention decreased the concentration of 5-HIAA by about 50% (3.4 vs. 6.3) and QA by about 45% (3.97 vs. 7.20). These changes were correlated with a significant improvement in the profitable action of budesonide on gastrointestinal and mental health of LC patients as they displayed significantly lower GSRS, HAM-A and HAM-B scores after than before the intervention—10.5 vs. 32, 11.0 vs. 21 and 12 vs. 18, respectively. In conclusion, a reduction in TRP intake in diet may improve GI and mental symptoms in LC patients treated with budesonide and these changes may be mediated by the products of TRP metabolism.

## 1. Introduction

Lymphocytic colitis (LC) is a chronic disease of the gastrointestinal (GI) tract characterized by chronic non-bloody diarrhea, cramping abdominal pain and other GI complaints [1]. It is an emerging problem for affected individuals, as this disease is associated with many symptoms which significantly decrease the patient’s quality of life, including severe abdominal pain, diarrhea and mood disorders. The pathogenesis of LC is poorly known but includes the immune reaction to many influences [2]. Recognized risk factors are advanced age, coexistence of autoimmune diseases, family history of inflammatory bowel disease (IBD), smoking and many drugs such as pain relievers, proton pump inhibitors and others; there are several putative risk factors, including dietary habits [3]. Therefore, proper nutrition may be an important factor to alleviate unpleasant LC-related symptoms lowering the quality of life. Another question is whether nutrition may assist in the pharmacotherapy of LC.

In most LC patients, colonoscopy shows a normal colonic mucosa, but vascular pattern and patchy erythema may be observed [4]. The main histological finding is the presence of at least 20 intraepithelial lymphocytes (IELs) per 100 epithelial cells, and this is the diagnostic criterion for this disease [4]. Nevertheless, in incomplete (borderline, paucicellular) cases, fewer number of IELs are accepted [5]. Apart from IELs, plasma cells, eosinophils, mast cells, macrophages and neutrophils are observed in the lamina propria [6]. The course of LC is usually mild, with periods of exacerbation and remission [7,8]. Reactive IELs may be involved in the activation of innate and acquired immunity and the secretion of many cytokines [9]. They are also thought to produce serotonin [10]. Moreover, IELs can migrate through the intestinal wall. Such two-way movement between the circulation and lymphatic tissue is needed for appropriate operation of the immune system [11]. It can be assumed that local immune processes are triggered as a result of active lymphocytes migrated from the GI tract to other organs, such as the lungs, thyroid gland, muscles and nervous system. Therefore, LC can be regarded as a general immune and inflammatory disease.

Depending on LC progression, there are various coexisting clinical manifestations [12]. For this reason, anti-inflammatory and immune-modulating drugs are used in the treatment of this disease. Currently, oral budesonide to achieve remission is endorsed as the first-line therapy. Other corticosteroids, thiopurines and biologicals may be used in selected patients who fail to respond to budesonide. Mesalazine, antibiotics and probiotics are not recommended [13,14].

In addition to pharmacological treatment in LC, a proper diet is also important. So far, the exact principles of nutritional treatment have not been established. It is generally recommended to limit the intake of saturated fats, carbohydrates and gluten-rich foods and to supplement some vitamins and maintain water and electrolyte balance [15,16]. Recently, the low FODMAP diet was applied, but the results obtained were not consistent. Some studies report an improvement in symptoms and inflammatory markers [17,18], while others do not testify any effect on abdominal pain, stool consistency and mucosal inflammation [19,20]. The above studies concerned ulcerative colitis and Crohn’s disease and did not specify LC.

L-tryptophan (TRP) is an essential amino acid provided with the diet or diet supplements. After intake, it is built into proteins or metabolized in three metabolic pathways—the kynurenine, serotonin and indole pathways—and the GI tract is the key location of TRP metabolism [21]. Impairment in TRP metabolic pathways is reported in many human diseases, including gastroenterological and mental disorders and is therefore targeted in dietary and pharmacological interventions [22,23]. The KYN pathway, which is responsible for about 95% of TRP metabolites, occurs mainly in epithelial and immune cells, and its metabolites play an important role in the regulation of the gut–brain–microbiota axis [24]. In our previous study in patients with LC, we found an increase in the number of enterochromaffin cells (ECs) and in the activity of tryptophan hydroxylase (TPH-1) in colonic mucosa, as well as an increase in serum serotonin levels [25]. In our next study, we found improvement in abdominal and mental symptoms after reducing TRP intake in patients with diarrhea-predominant irritable bowel syndrome, as well as beneficial changes in metabolism [26]. The above results inspired further research on the role of this amino acid in the pathogenesis of LC. The aim of the present study was to assess the impact of a low-TRP diet on the abdominal symptoms and mental state of patients with LC treated with budesonide.

## 2. Materials and Methods

### 2.1. Patients

One hundred and twenty individuals were recruited to this study in 2018–2022 in the Department of Clinical Nutrition and Gastroenterological Diagnostics and in the Department of Gastroenterology, Medical University of Lodz, Lodz, Poland. The control group included 40 individuals without any complaints. Exclusion criteria were organic diseases of the GI tract other than LC, *H. pylori* infection, small intestinal bacterial overgrowth (SIBO), allergy and food intolerance, parasitic and bacterial diseases, liver and renal diseases, diabetes and severe anxiety or depression. The patients were asked to report all the drugs they used, and those who reported taking antibiotics, probiotics and psychotropic drugs in the month prior to enrolment into the study were excluded. The patients group was recruited in two stages. Firstly, a population of individuals suffering from loose or watery stools from 14 months to 6 years were initially classified as “general patients”. The number of loose stools ranged from 3 to 8 a day and did not coexist with occasional constipation. These patients suffered from abdominal pain related to defecation, bloating and flatulence. In addition, patients complained of anxiety, depressed mood and sleep disturbances. Then, the number of colonic IELs per 100 epithelial cells was determined (Section 2.2) and those “general patients” who had 20 or more IELs were classified as LC patients.

### 2.2. Laboratory and Clinical Examinations

#### 2.2.1. Routine Laboratory Tests

To determine if there is a difference in basic biochemical parameters between controls and LC patients, the following laboratory tests were performed in all subjects: blood cell count, quantification of protein, glucose, glycated hemoglobin, profile of lipids, bilirubin, iron, urea, creatinine, thyroid stimulating hormone, free thyroxine, free triiodothyronine antibodies to tissue transglutaminase, deaminated gliadin peptide, as well as the activity of alanine and asparagine aminotransferase, alkaline phosphatase, gamma-glutamyltranspeptidase, amylase and lipase, deaminated gliadin peptide, C-reactive protein (CRP) and fecal calprotectin (FC).

#### 2.2.2. Lymphocytic Colitis Diagnosis

The presence of ILEs was detected by hematoxylin and eosin staining. Lymphocytic colitis was diagnosed when the number of IELs in colonic biopsies was equal to or exceeded 20 per 100 epithelial cells. As colonic enterochromaffin cells (ECs) are involved in TRP metabolism, their number was determined with mouse monoclonal antibodies (chromogranin A-LK2H, Cell Marque Co., Hague, The Netherlands) and the UltraVision Quanto Detection System (HRP-DAB, Immunologic BV, Duiven, The Netherlands) in the range 10 fields in each biopsy specimen at 40× magnification.

#### 2.2.3. Patients’ Self-Assessment of Gastrointestinal and Mental Symptoms

To correlate GI disorders with budesonide treatment and dietary intervention the LC patients were asked to answer questions on the severity of abdominal symptoms, which was assessed with the Gastrointestinal Symptom Rating Scale—Irritable Bowel Syndrome (GSRS-IBS) [23]. All patients self-assessed their psychological state, and then everyone was evaluated with the Hamilton Anxiety Rating Scale (HAM-A) and the Hamilton Depression Rating Scale (HAM-D) [27].

#### 2.2.4. Determination of Tryptophan and Its Metabolites in Urea

To evaluate an influence of pharmacological and dietary intervention on TRP metabolism, TRP and its following metabolites in urine were determined: kynurenine (KYN), kynurenic acid (KYNA) and quinolinic acid (QA). Urine samples were taken on empty stomach, and TRP metabolites were determined by liquid chromatography with tandem mass spectrometry (Ganzimmun Diagnostics AG, Mainz, Germany).

### 2.3. Therapeutic Interventions

All subjects recorded all components of their diet two weeks before start of the study. TRP intake was evaluated by the nutritional calculator with the application Kcalmar.pro-Premium (Hermex, Lublin, Poland). All subjects had a balanced diet. On the first day of the study, each subject was given a diet with the TRP content calculated in advance and such diet was applied for 8 weeks. All LC patients received budesonide at a daily oral dose of 9 mg for 8 weeks. The patients were allocated into two groups of 40 persons each. One group had a balanced diet with the normal content of TRP (LC TRP norm) and the other group had a diet with a 25% reduced TRP intake (LC TRP–). The use of any drugs was forbidden. 

The research was conducted as an open-label clinical trial according to the guidelines of the Declaration of Helsinki and the Guidelines for Good Clinical Practice and was approved by the Bioethics Committee of Medical University of Lodz (RNN/176/18/KE).

### 2.4. Data Analysis

Shapiro–Wilk W test was employed to verify data distribution. The Student’s *t*-test, Welch F test and U Mann–Whitney’s test compared differences between groups. Differences in pre- and post-treatments were checked by Wilcoxon signed-rank test. Sample size was calculated using Sample Size Calculator within Statistica 13.3 software. Setting α = 0.05 (bilateral), β = 0.10 and the number of groups k = 3, the minimum sample size of 34 cases per group was calculated. The internal consistency of questionnaires was calculated using Cronbach’s alpha. The value of overall internal consistency of questionnaires, expressed by Cronbach’s alpha were around 0.65, which is considered acceptable. All statistical analyses were performed with Statistica 13.3 software (TIBCO Software Inc., Palo Alto, CA, USA).

## 3. Results

Table 1 presents some basic characteristics of individuals enrolled in this study before pharmacological and dietary interventions. The LC patients group contained almost equal numbers of males and females, whereas females dominated in the control group (*p* < 0.001). The concentrations of CRP and FC as well as the number of IELs and ECs were higher in LC patients than in controls (*p* < 0.001).

Table 2 presents the values of urinary concentrations of TRP and its metabolites in controls and LC patients before pharmacological and dietary interventions.

LC patients had had lower urinary concentrations of TRP, while the concentrations of 5-HIAA, KYN and QA were higher than in the controls.

Budesonide treatment decreased GI complaints in LC patients, and dietary intervention with reduced TRP further decreased GI syndromes (Table 3). Likewise, budesonide treatment improved mental disturbances in LC patients, and dietary TRP-based intervention further improved the mental state of LC patients. Intertreatment observations made after 4 weeks of therapy and dietary intervention showed that diarrhea was completely resolved in about 40% of LC patients without dietary intervention and about 77% with dietary intervention. Similarly, a significant improvement in mood disorders was observed in the first four weeks of budesonide therapy, and dietary intervention strengthened these changes.

Table 4 presents data on the effect of the TRP-based 8-week dietary intervention on the urine concentration of TRP and its main metabolites in the kynurenine pathway in LC patients treated with budesonide for 8 weeks. The dietary intervention significantly reduced the concentration of TRP, 5-HIAA and QA, but it increased KYNA concentration.

It is not surprising that a TRP-reduced diet decreased urine TRP concentration, but the products of TRP metabolism were differentially affected by the dietary intervention. The changes in the concentration of KYNA deserves a special attention, as it was lower in the LC patients undergoing dietary intervention than in LC patients without such intervention. However, after completing the intervention, KYNA concentration increased and was greater than in LC patients without intervention.

We also determined the concentrations of biochemical indicators that were different in LC patients and controls: CRP and FC. No changes were observed in these parameters with dietary intervention with reduced TRP content in LC patients (data not shown).

## 4. Discussion

Our study can be classified as an open-label trial mode. All subjects either received compound(s) of interest or not. There was not a placebo group, because our study was based on changes in the diet and not a diet supplementation.

Both kinds of the diet were tolerated well in LC patients. However, limited consumption of some products such as white bread, pasta and processed meat was not always gratefully accepted. The patients did not complain of any unwanted effect. Compliance and cooperation between patients and dietitians were excellent.

The general characteristics of the subjects enrolled in this study (Table 1) indicated that the LC patients and controls differed only in parameters related to LC: IEL and EC numbers, CRP and FC concentrations. All other biochemical and demographic parameters did not differ between those groups, except for gender. We focused on GI syndromes (first stage) and IEL number (second stage) during the recruiting procedure and did not include gender selection, as it would have significantly limited the number of patients or extended the time of the experiment. We do not draw any conclusion on this gender imbalance, as it may be influenced by several factors that are not completely recognized and require further research.

It seems paradoxical that we introduced a diet with lowered TRP content when LC patients had lower TRP levels than the controls (Table 2). However, LC patients showed higher levels of two main TRP metabolites in the KYN pathway, 5-HIAAA and QA. The concentration of these two compounds decreased after dietary intervention (Table 3). We therefore conclude that not TRP itself but the products of its metabolism, including 5-HIAA and QR, may contribute to the improvement in budesonide treatment and alleviation in mental state of LC patients. However, we did not investigate the product of the two remaining pathways of TRP metabolism, the serotonin and indole pathways, which may also contribute to these effects. Therefore, reduction in dietary TRP intake resulted in improvement in GI complaints related to the beneficial action of budesonide and the mitigation of mental syndromes in LC patients. These improvements were positively correlated with a decrease in the concentration of two main TRP metabolites in the KYN pathway, 5-HIAA and QA (Table 3 and Table 4).

The obtained results confirm previous findings indicating that during the exacerbation of LC, serotonin secretion is increased [25]. The main source of serotonin are EC cells, whose number increases in the active phase of other inflammatory bowel diseases [27,28]. Excess serotonin impairs the motor and secretory function of the intestines, causing diarrhea and abdominal pain and inducing a pro-inflammatory effect [29]. Pharmacological treatment using various anti-inflammatory agents results in histological and clinical remission, but usually does not provide long-term remission [30,31,32]. Studies on the restriction or enrichment of TRP intake were conducted mainly in patients with mental disorders [33,34,35,36]. An increase in dietary TRP resulted in augmentation of depressive symptoms and decreased anxiety [37,38]. In these clinical trials, TRP was supplemented in various doses, significantly exceeding the physiological requirement for this amino acid. Too high doses of TRP may cause changes in both the serotonin and kynurenine pathways of its metabolism, as well as result in unwanted side effects [39]. In such situations, an excess of neurotoxic compounds can be formed, resulting in depressive symptoms [40]. In LC patients, both serotonin, as marked by 5-HIAA and kynurenine pathways, as indicated by KYN, KYNA and QA, showed higher activity compared to healthy individuals. The increase in serotonin synthesis is probably a consequence of an increase in the number of EC cells. In addition, inflammatory factors, such as cytokines, and glucorticosteroids used in long-term therapy of LC could stimulate IDO-1, which initiates the kynurenine pathway [41]. This may be the reason for the frequent coexistence of mental disorders in bowel inflammatory diseases.

The worldwide consumption of TRP depends on local dietary habits and averages around 800–1200 mg per day [41]. Our LC patients consumed a daily average of 1309 mg of TRP initially and 814 mg during the dietary intervention. Such intake did not result in negative effects on general health, but further research should aim to establish an optimal TRP intake in long-term therapy and in the remission of LC. In addition, in these studies, it is necessary to standardize the criteria for disease activity. The usefulness of many biomarkers has been suggested so far, such as fecal eosinophil protein, eosinophil cationic protein, fecal lactoferrin, alpha-2-antitriptin, tryptase, myeloperoxidase and others, but they have not been widely accepted. Fecal calprotectin is also not useful to exclude or monitor LC [42], which is also confirmed by our research. Although FC concentrations were slightly higher than in the control group, they did not change after treatment. Control colonoscopy and histological examination is not recommended because, in many cases, including borderline and paucicellular cases, the microscopic picture does not correlate with clinical activity [43]. In our previous and current studies, we found a positive correlation between urinary 5-HIAA excretion and the severity of symptoms in LC patients. Therefore, urinary excretion of 5-HIAA may be a useful non-invasive biomarker. Various methods have also been used to clinically assess LC activity. Neither the Hjortswang criteria nor the MC Diseases Activity Index (MCDAI) have undergone formal prospective validation requirements from the Food and Drug Administration for patients’ reported outcomes in clinical trials [32]. For this reason, the criteria adopted for IBS were also used. We adopted criteria based on studies of the European population, considering not only the frequency of bowel movements, but also other abdominal complaints. Some of them, such as visceral pain or urgent need for bowel movement, such as diarrhea, significantly worsen the quality of life of patients.

Our study is not free from limitations. The results of LHBT do not exclude all species of bacteria with a potential to colonize the colon. These bacteria may influence the TRP metabolism [44,45]. Several strains of bacteria can metabolize TRP and convert it into indole and skatole, which are reported to activate the immune system, stimulate GI motility and secretion of intestinal hormones [46,47]. However, the main limitation of our study is to consider only one pathway of TRP metabolism, the KYN pathway. The two remaining pathways, the serotonin and indole pathways, may be also affected by a diet with reduced TRP content. Therefore, studies on the serotonin and indole pathways of TRP metabolism in patients with LC and mood disorders seem to be a close perspective to complete our current research.

To our knowledge, there is no other study on dietary intervention with TRP intake in LC. Searching on PubMed with the entry “lymphocytic colitis tryptophan” returned three items. One of them does not deal with LC, but with inflammatory bowel disease [48]. The remaining two are works performed by our team and neither deals with TRP or its metabolites in the KYN pathway; instead, they consider serotonin and melatonin [25,28]. These works were discussed above. Therefore, we cannot directly compare our results with any other results as, so far, no study on TRP-based dietary intervention in LC patients has been reported. In this light, we can consider our study as a pioneering work.

In summary, reduced TRP intake may improve budesonide action, reducing gastrointestinal and mental complaints in lymphocytic colitis patients.

## Figures and Tables

**Table 1:** Characteristics of control individuals (n = 40) and patients with lymphocytic colitis (LC, n = 60) enrolled in this study.

Feature ^a^	Controls	LC Patients
Age (years)	46.1 ± 10.2	48.3 ± 9.94
Gender (M/F)	8/32	27/33 ***
BMI (kg/m^2^)	23.4 ± 1.9	22.9 ± 1.85
GFR (mL/min)	94.6 ± 5.32	96.5 ± 4.96
AST (U/L)	13.2 ± 1.93	15.1 ± 2.55
ALT (U/L)	15.8 ± 3.51	17.6 ± 4.21
CRP (µg/L)	2.42 ± 0.89	3.56 ± 0.91 ***
FC (µg/g)	26.8 ± 7.81	37.4 ± 7.88 ***
IEL (no.)	13.9 ± 3.52	31.8 ± 5.76 ***
EC (no.)	26.8 ± 7.81	32.4 ± 7.85 ***
TRP intake (mg/kg bw/d)	20.8 ± 2.46	21.3 ± 2.16

^a^ mean ± SD (standard deviation); BMI—body mass index; CRP—C-reactive protein; FC—fecal calprotectin; AST—aspartate aminotransferase; ALT—alanine aminotransferase; GFR—glomerular filtration rate; IEL—intraepithelial lymphocyte; EC—enterochromaffin cell; TRP—tryptophan; differences between groups were assessed by Student’s *t*-test, ***—*p* < 0.001.

**Table 2:** Concentration of tryptophan (TRP) and main products of its metabolism in the kynurenine pathway in urine of controls (*n* = 40) and lymphocytic colitis (LC) patients (*n* = 60) enrolled in this study.

Product ^a^	Controls	LC Patients
TRP	13.78 ± 1.851	11.54 ± 1.225 ***
5-HIAA	2.93 ± 0.96	6.54 ± 0.76 ***
KYN	0.46 ± 0.11	0.61 ± 0.18 ***
KYNA	2.39 ± 0.50	2.37 ± 0.71
QA	3.07 ± 0.94	7.11 ± 1.03 ***

^a^ mean ± SD (standard deviation); all concentrations were expressed in mg per gram of creatinine (mg/g CR); TRP—tryptophan; 5-HIAA—5-hydroxyaminoacetic acid; KYN—kynurenine; KYNA—kynurenic acid; QA—quinolinic acid; differences between groups were assessed either by Student’s *t*-test or Welch F-test, which was used when variances in two groups were not equal (TRP and KYN); ***—*p* < 0.001.

**Table 3:** Severity of gastrointestinal complaints and mental disturbances in lymphocytic colitis (LC) patients with normal and reduced dietary TRP content (TRP norm and TRP–, respectively; *n* = 30 in either group) before or after 8 weeks of budesonide treatment at 9 mg daily.

Symptom Rating Scale ^a^	Budesonide Treatment	LC TRP Norm	LC TRP–
GSRS	before	31.5 (6.0)	31.0 (6.0)
	after	14.0 (4.0)	10.5 (3.0) ***
HAM-A	before	19.5 (3.0)	21.0 (4.0)
	after	14.0 (5.0)	11.0 (3.0) **
HAM-B	before	19.0 (7.0)	18.0 (7.0) **
	after	16.0 (5.0)	12.0 (2.2) ***

^a^ median (IQR, interquartile range); GSRS—Gastrointestinal Symptom Rating Scale—Irritable Bowel Syndrome; HAM-A—Hamilton Anxiety Rating Scale; HAM-D—Hamilton Depression Rating Scale; differences between groups with and TRP-based dietary intervention were assessed by the Wilcoxon matched-pairs signed-rank test; **—*p* < 0.01; ***—*p* < 0.001; differences between before and after budesonide were significant at *p* < 0.001 for each symptom rating scale.

**Table 4:** Concentration of tryptophan (TRP) and main products of its metabolism in the kynurenine pathway in urine of LC patients treated for 8 weeks with budesonide at 9 mg daily without or with dietary intervention with reduced TRP (LC TRP norm and LC TRP–, respectively; *n* = 30 in each group) before and after budesonide treatment.

Product ^a^	Budesonide Treatment	LC TRP Norm	LC TRP–
TRP	before	11.550 (2.00)	11.300 (1.70)
	after	11.300 (1.90)	9.800 (1.00) ***
5-HIAA	before	6.600 (1.00)	6.300 (1.60)
	after	5.650 (0.80)	3.400 (0.70) ***
KYN	before	0.630 (0.26)	0.560 (0.15)
	after	0.550 (0.28)	0.515 (0.15)
KYNA	before	2.560 (0.73)	2.130 (0.64) **
	after	2.735 (0.65)	3.165 (0.86) **
QA	before	7.240 (1.76)	7.200 (1.93)
	after	6.920 (1.85)	3.970 (1.16) ***

^a^ median (IQR, interquartile range); all concentrations were expressed in mg per gram of creatinine (mg/g CR); TRP—tryptophan; 5-HIAA—5-hydroxyaminoacetic acid; KYN—kynurenine; KYNA—kynurenic acid; QA—quinolinic acid; differences between groups were assessed by Mann–Whitney U test; **—*p* < 0.01, ***—*p* < 0.001.

## Data Availability

Not applicable.
